# The Benzimidazole Derivatives, B1 (*N*-[(1*H*-Benzimidazol-2-yl)Methyl]-4-Methoxyaniline) and B8 (*N*-{4-[(1*H*-Benzimidazol-2-yl)Methoxy]Phenyl}Acetamide) Attenuate Morphine-Induced Paradoxical Pain in Mice

**DOI:** 10.3389/fnins.2019.00101

**Published:** 2019-02-12

**Authors:** Zahida Idris, Muzaffar Abbas, Humaira Nadeem, Arif-ullah Khan

**Affiliations:** ^1^Department of Basic Medical Sciences, Riphah Institute of Pharmaceutical Sciences, Riphah International University, Islamabad, Pakistan; ^2^Department of Pharmaceutical Chemistry, Riphah Institute of Pharmaceutical Sciences, Faculty of Pharmaceutical Sciences, Riphah International University, Islamabad, Pakistan

**Keywords:** paradoxical pain, TNF-α, mice, morphine, benzimidazole derivatives

## Abstract

Despite being routinely used for pain management, opioid use is limited due to adverse effects such as development of tolerance and paradoxical pain, including thermal hyperalgesia and mechanical allodynia. Evidence indicates that continued morphine administration causes increased expression of proinflammatory mediators such as tumor necrosis factor-alpha (TNF-α). The objectives of the present study were to determine the effects of B1 (*N*-[(1*H*-benzimidazol-2-yl)methyl]-4-methoxyaniline) and B8 (*N*-{4-[(1*H*-benzimidazol-2-yl)methoxy]phenyl}acetamide), benzimidazole derivatives, on thermal nociception and mechanical allodynia during repeated morphine (intraperitoneal; 5 mg/kg twice daily for 6 days)-induced paradoxical pain and TNF-α expression in the spinal cord in mice. Our data indicate that administration of benzimidazole derivatives attenuated morphine-induced thermal hyperalgesia and mechanical allodynia. Benzimidazole derivatives also reduced TNF-α expression in mice. Taken together, these results suggest that benzimidazole derivatives might be useful for the treatment of neuroinflammatory consequences of continued morphine administration and could be potential drug candidates for the management of opioid-induced paradoxical pain.

## Introduction

Pain is a significant social, clinical, and economic health problem (Phillips, 2006). It usually results from activation of nociceptive afferents by actual or potential tissue-damaging stimuli (Treede et al., 2008). Pain may be acute or chronic depending on disease status (Muscoli et al., 2007). A research report indicates that the prevalence of chronic pain ranges from 8 to 60% (Phillips, 2006).

Opioids such as morphine remain the drug of choice in alleviating moderate to severe pain (Johnston et al., 2004). At present, the prescribing of opioids is increasing in the United States. However, long-term opioid treatment is associated with reduced clinical efficacy and paradoxical pain development characterized by hyperalgesia and allodynia in humans and in experimental animals (Tompkins and Campbell, 2011). It has been reported that the prolonged use of morphine may result neuro-inflammation in the central nervous system and hyperalgesia that leads to the suppression of morphine analgesia (Wang et al., 2012).

Opioid-induced hyperalgesia in animals can be defined as a paradoxical state of heightened pain sensation in which both pain threshold and pain tolerance decrease from baseline after chronic administration of opioids (Stoicea et al., 2015). Meanwhile, allodynia can be defined as the experience of pain from a benign stimulus (Tompkins and Campbell, 2011). The exact mechanism underlying opioid-induced paradoxical pain is still unknown (Tumati et al., 2012). However, it has been studied that activation of glial cells after sustained morphine administration increases the expression of proinflammatory mediators such as tumor necrosis factor-alpha (TNF-α), which contributes to the development of opioid-induced hyperalgesia and allodynia (Sommer and Kress, 2004; Stellwagen and Malenka, 2006). Both glial cell (microglia and astrocytes) activation and enhanced proinflammatory cytokines including TNF-α expression were observed following chronic morphine treatment at the spinal cord of the rodents (Raghavendra et al., 2002). The TNF-α released in response to various insults and injury (Merrill and Benveniste, 1996) produces its effect by rapidly increasing pain sensation (Beattie et al., 2002). These facts indicate that TNF-α plays a critical role in paradoxical pain development (Grace et al., 2015) and decreasing TNF-α expression might be helpful in reducing morphine-induced paradoxical pain. Thus, inhibition of glial cell activation or antagonizing the activity of TNF-α might attenuate the development of morphine-induced hyperalgesia in laboratory animals (Raghavendra et al., 2002).

Benzimidazole is an important scaffold having different biological activities. Benzimidazole moieties have benzene and a heterocyclic imidazole ring, and are one of the most promising moieties that is present in many clinically useful drugs (Salahuddin et al., 2017). Previous studies showed that benzimidazole derivatives have considerable anti-inflammatory and analgesic properties (Akhtar et al., 2017). We therefore hypothesized that systemic administration of benzimidazole derivatives with morphine might be able to reduce opioid-induced hyperalgesia and allodynia.

**FIGURE 1:**
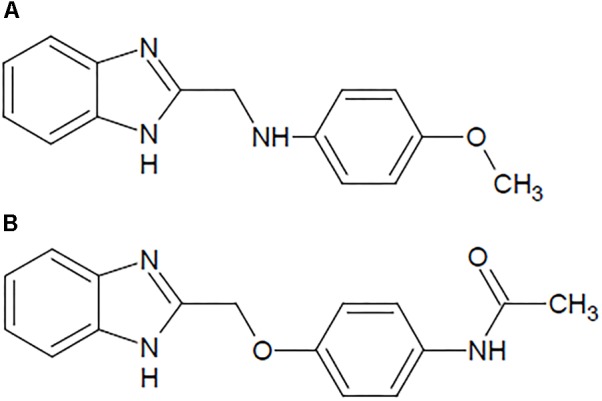
Structure of the benzimidazole derivatives: **(A)** *N*-[(1*H*-benzimidazol-2-yl) methyl]-4-methoxyaniline (B1) and **(B)** *N*-{4-[(1*H*-benzimidazol-2-yl) methoxy] phenyl} acetamide (B8).

The present study was designed to evaluate the possible beneficial effects of benzimidazole derivatives B1 (*N*-[(1*H*-benzimidazol-2-yl)methyl]-4-methoxyaniline) and B8 (*N*-{4-[(1*H*-benzimidazol-2-yl)methoxy]phenyl}acetamide), shown in (Figure 1) against repeated morphine administration-mediated thermal hyperalgesia, tactile allodynia, and on spinal TNF-α expression in an animal model. We also explored the antioxidant potential and the acute toxicity of the selected benzimidazole derivatives.

## Materials and Methods

### Animals

Balb-c mice of either gender (25–30 g), equal in number for groups with an even number of animals and with one more male mouse in groups with an odd number of animals, were used for experimental work. Animals were housed in groups of four in standard cages (22 ± 2°C, relative humidity of between 50 and 60%), allowing free access to diet and water, and maintained on a reverse 12/12 h light/dark cycle at the animal house of the Riphah Institute of Pharmaceutical Sciences (RIPS) (Council, 1996). The experimental procedures were approved by RIPS Ethical Committee, Pakistan (Ref No. REC/RIPS/2016/014).

### Chemicals

All drug doses were calculated on the basis of animal weight. Morphine was procured from Sigma Aldrich through proper channels and was diluted in normal saline (0.9% NaCl). The benzimidazole derivatives, B1 and B8, were synthesized at the Department of Chemistry, Riphah Institute of Pharmaceutical Sciences, Riphah International University, Islamabad, Pakistan, and were diluted in normal saline containing 5% DMSO and 2.5% Tween 80. The TNF-α mouse ELISA (ab100747) kit was purchased from Abcam.

### Drug Administration

**FIGURE 2:**
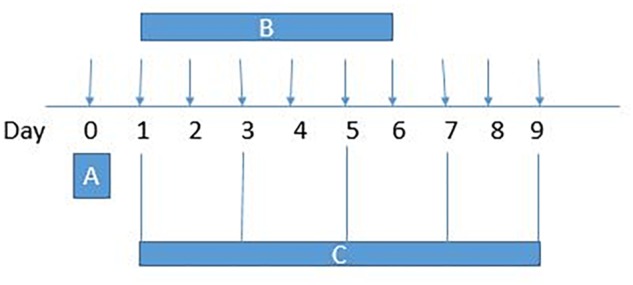
Experimental design: **(A)** On day 0, each mouse baseline behavioral test – paw withdrawal latency (hotplate test), tail withdrawal latency (tail immersion test) and paw withdrawal threshold (von Frey filament test) – was measured. **(B)** Animals received intraperitoneal vehicle or vehicle+morphine (5 mg/kg) or B1/B8 (1 mg/kg, 3 mg/kg, or 9 mg/kg)+morphine (5 mg/kg) or ketamine (10 mg/kg)+morphine (5 mg/kg); injected twice daily for 6 days (Day 1–Day 6). **(C)**The animals were tested for paw withdrawal latencies, tail withdrawal latency and paw withdrawal thresholds every other day (1st, 3rd, 5th, and 7th day at 08:30 h) during the drug treatment period 30 min after morphine/saline injection and 96 h (9th day) after the last drug injection.

Paradoxical pain was induced with morphine (5 mg/kg/injection, 10 ml/kg volume), delivered intraperitoneally (IP), twice daily (08:00 h and 20:00 h) for 6 days (*n* = 5–6 animals). Separate groups of animals received IP injections of B1 (1, 3, or 9 mg/kg) twice daily along with morphine for 6 days (*n* = 5–6 days). Another separate group of animals received IP injections of B8 (1, 3 or 9 mg/kg) twice daily along with morphine for 6 days (*n* = 5–6). Animals received benzimidazole derivatives 30 min before morphine injection. Negative control animals received an equal volume of vehicle (10 ml/kg). Positive control group animals received ketamine (10 mg/kg) along with morphine (Laulin et al., 2002). Behavioral (thermal nociception and mechanical allodynia) assays were blindly performed prior to drug administration (naïve baseline), every alternate day (1st, 3rd, 5th, and 7th day at 08:30 h) during drug treatment period 30 min after morphine/saline injection and at 96 h (9th day) after the last IP drug injection (Figure 2).

### Thermal Hyperalgesia

Thermal hyperalgesia was performed using a hot plate assay (Yoon et al., 2012). Briefly, each animal in a plexiglass chamber was positioned on a hot plate maintained at 55°C ± 2. Paw withdrawal latency was measured by using a stopwatch. The time spent by animals to exhibit licking, flicking or jumping was recorded as a positive response. A maximal 30 s cutoff was used to prevent tissue damage.

### Mechanical Allodynia

Mechanical allodynia was performed using von Frey filaments as described previously (Chaplan et al., 1994). Briefly, mice were allowed to habituate to the experimental apparatus for 30 ± 5 min in wire mesh cages. Using an up-and-down method, calibrated Von Frey filaments of 0.16, 0.4, 0.6, 1, 1.4, and 2 g of different strength were used in accordance with the up-and-down method. A starting filament with 0.16 g was applied perpendicularly to the plantar surface of the mice paw kept in wire-mesh cages. Positive score was recorded as paw withdrawal and next lighter filament was applied. For negative scoring, next higher force filament was used. The same procedure was continued for five consecutive readings (Xie et al., 2005; Tumati et al., 2012). The average of five scores was recorded.

### Thermal Nociceptive Test

Thermal nociceptive susceptibility was measured with the thermal nociceptive tail withdrawal test as described previously (Johnston et al., 2004). Briefly, mice were placed in individual plastic tubes with tail lying outside. The lower 5 cm allocated section of the mice tail was dipped in a water bath maintained at 45°C. Withdrawal of the tail within a few seconds was recorded by stopwatch as a positive response. A cutoff time of 10 s was used to avoid tail damage (Xie et al., 2005).

### Enzyme-Linked Immunosorbent Assay

An enzyme-linked immunosorbent assay (ELISA) was used to measure TNF-α in the spinal cord of mice. Briefly, 96 h after the last IP dose of morphine, the animals were sacrificed and their lumber spinal cords were isolated. The spinal tissues were stored at -80°C until analysis. On the day of experiment, the tissues were homogenized by a probe sonicator in tris buffer saline containing 5% Tween 80, centrifuged at 8500 × *g* for 30 min at 4°C, and their supernatants were collected (Tumati et al., 2010). The mouse TNF-α ELISA kit (ab100727) was used for TNF-α quantification as per manufacturer’s instructions.

### Antioxidant Activity

Antioxidant activity of the selected benzimidazole derivatives was measured using a 2-diphenyl-1-picrylhydrazyl (DPPH) radical scavenging assay (Molyneux, 2003; Mathew and Abraham, 2006). Serial dilutions of the tested compounds were prepared with a concentration of 1, 3, 10, 100, 300, 700, and 100 μg/ml in 5% DMSO and 2.5% Tween 80. Then, 3 mL from the freshly prepared 1 Mm DPPH solution in methanol was added to each dilution. After vigorous shaking, the mixture was placed in a dark place for 30 min to complete the reaction, showing a change in color. With a UV spectrophotometer, absorbance of the tested solution was measured at 517 nm. From the given formula, the percentage of DPPH inhibition from the sample was calculated.

(1) $$\mathrm{\%Inhibition}=\left\{\frac{\mathrm{ABScontrol}-\mathrm{ABSsample}}{\mathrm{ABS control}}\right\}\%100$$

A concentration (μM) versus % inhibition graph was plotted using Graph pad prism 6.0. The IC_50_ was calculated for each test compound. The experiment was also carried out without having tested compounds to serve as a negative control. The same experimental protocol was followed for ascorbic acid referred to serve as a positive control. All experiments were performed in triplicate (Molyneux, 2003).

### Determination of Acute Toxicity in Animal Model

Animals (*n* = 3) were divided into two groups. Group 1 received B1 (1000 mg/kg), whereas group 2 received B8 (1000 mg/kg). The animals were then observed for 24 h for their behavior and mortality (Chinedu et al., 2013).

### Data Analysis

The data were analyzed using the Graph Pad Prism 6.0 (Graph-Pad, San Diego, CA, United States). Behavioral data were analyzed by two-way ANOVA followed by *post hoc* Tukey’s test for multiple comparison. Meanwhile, TNF-α contents were analyzed by one-way ANOVA followed by Tukey’s *post hoc* test. Statistical differences were considered significant at *p* < 0.05 (^∗^*p* < 0.05; ^∗∗^*p* < 0.01, ^∗∗∗^*p* < 0.001). Data are presented as mean ± SEM unless otherwise indicated.

## Results

### Effect of Benzimidazole Derivatives on Repeated Morphine-Mediated Thermal Hyperalgesia

**FIGURE 3:**
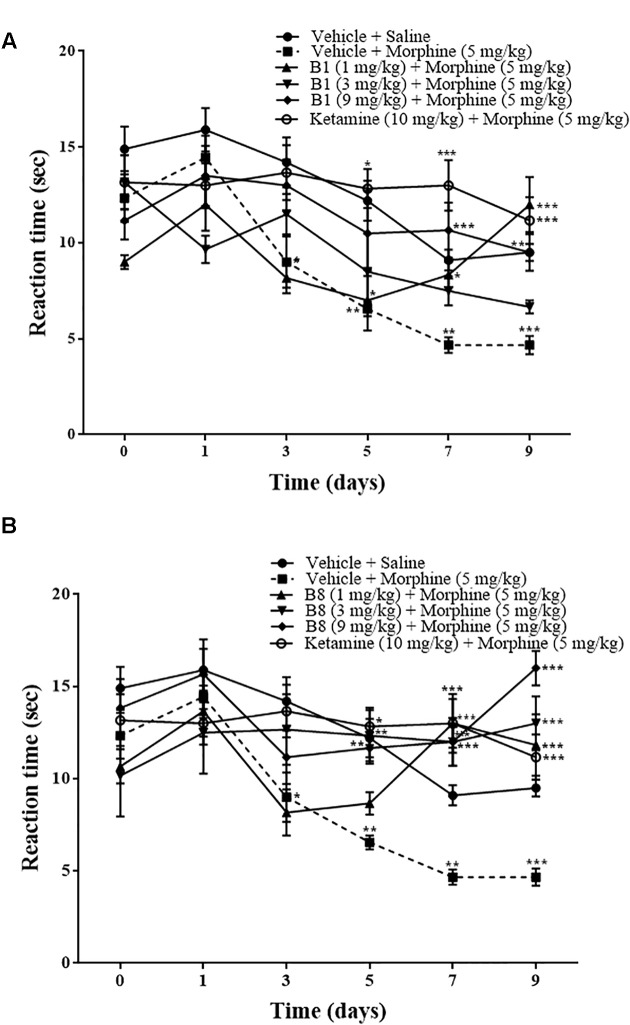
Effects of benzimidazole derivative, B1/B8, on morphine-induced thermal hyperalgesia in mice. Mice of either gender (*n* = 6–10) received intraperitoneal vehicle; vehicle+morphine (5 mg/kg); ketamine (10 mg/kg)+morphine; **(A)** B1 (1 mg/kg, 3 mg/kg, or 9 mg/kg)+morphine or **(B)** B8 (1 mg/kg, 3 mg/kg, or 9 mg/kg)+morphine injected twice daily for 6 days. Thermal pain sensitivity was measured performed prior to drug administration (naïve baseline), every alternate day (1st, 3rd, 5th, and 7th day at 08:30 h) during the drug treatment period 30 min after morphine/saline injection and 96 h (9th day) after the last IP morphine injection by using hotplate assay (54°C ± 1°C). Negative control animal received an equal volume of vehicle. Analyzed by two-way ANOVA followed by Tukey’s *post hoc* test. Data are expressed as mean ± SEM. ^∗^*p* < 0.05, ^∗∗^*p* < 0.01, ^∗∗∗^*p* < 0.001.

Continuous morphine treatment led to a gradual decrease in mean paw withdrawal latencies in hot plate tests and increased thermal hypersensitivity. The decrease in mean paw withdrawal latencies was significant starting from day 3 of morphine treatment (vehicle-morphine group, 9 ± 1.3 s; ^∗^*p* < 0.05 relative to vehicle-saline treated control group 14.2 ± 1.30 s, two-way ANOVA, *n* = 9) (Figure 3A,B). On day 5 and 7, mean paw withdrawal latencies of the vehicle-morphine group were 6.55 ± 0.37 s and 4.66 ± 0.408 s, ^∗∗^*p* < 0.01, two-way ANOVA relative to the vehicle-saline treated the negative control group. At 96 h after morphine withdrawal, there was a significant decrease in mean paw withdrawal latency (4.66 ± 0.47 s, ^∗∗∗^*p* < 0.001 relative to vehicle-saline negative control group, two-way ANOVA, *n* = 9) (Figure 3). Administration of the benzimidazole derivative B1 (1 mg/kg) attenuated decreases in paw withdrawal latency by repeated morphine administration, which were 7 ± 1.57 s (^∗^*p* < 0.05) on day 5, 8.33 ± 0.66 s (^∗^*p* < 0.05) on day 7, and 12 ± 1.43 s (^∗∗∗^*p* < 0.001) at 96 h after withdrawal, relative to the vehicle-morphine group (two-way ANOVA, *n* = 6) (Figure 3A). Paw withdrawal latency in mice treated with B1 (9 mg/kg) was 10.66 ± 1.4 s (^∗∗∗^*p* < 0.001) on day 7, and 9.5 ± 96 s (^∗∗^*p* < 0.01) at 96 h after withdrawal, relative to the vehicle-morphine group (two-way ANOVA *n* = 6) (Figure 3A). Mean paw withdrawal latency of the compound B8 (3 mg/kg) was 12.33 ± 1.38 s (^∗∗^*p* < 0.01) on day 5, 13 ± 1.59 s (^∗∗∗^*p* < 0.001) on day 7, and 13 ± 1.46 s (^∗∗∗^*p* < 0.001) at 96 h after withdrawal vs. the vehicle-morphine treated group (two-way ANOVA, *n* = 6) (Figure 3B). Mean paw withdrawal latency of the B8 (9 mg/kg) was 11.66 ± 0.84 s (^∗∗^*p* < 0.01) on day 5 relative to the vehicle-morphine treated group (two-way ANOVA, *n* = 6) (Figure 3B). While mean paw withdrawal latency of the B8 (1 mg/kg or 9 mg/kg) was 12.83 ± 1.13 s (^∗∗∗^*p* < 0.001) or 12 ± 1.2 s (^∗∗∗^*p* < 0.001) on day 7, and 11.83 ± 1.66 s (^∗∗∗^*p* < 0.001) or 16 ± 0.93 s (^∗∗∗^*p* < 0.001) at 96 h after withdrawal, relative to the vehicle-morphine treated group (two-way ANOVA, *n* = 6) (Figure 3B). Administration of ketamine (10 mg/kg) attenuated repeated morphine-mediated decreases in paw withdrawal latencies. The mean paw withdrawal latency of the mice treated in the morphine-ketamine positive control group was 12.83 ± 1.01 s (^∗^*p* < 0.05) on day 5, 13 ± 1.31 s (^∗∗∗^*p* < 0.001) on day 7, and 11.16 ± 1.22 s (^∗∗∗^*p* < 0.001) at 96 h after withdrawal, relative to the vehicle-morphine group (two-way ANOVA, *n* = 6) (Figure 3A,B). Overall, these results showed that repeated treatment with morphine resulted in a decrease in paw withdrawal latency over the time, indicating a gradual development of sensitivity to thermal stimuli. However, co-administration of benzimidazole derivatives reduced thermal hyperalgesic effects of repeated morphine administration.

### Effects of Benzimidazole Derivatives on Repeated Morphine-Mediated Mechanical Allodynia

**FIGURE 4:**
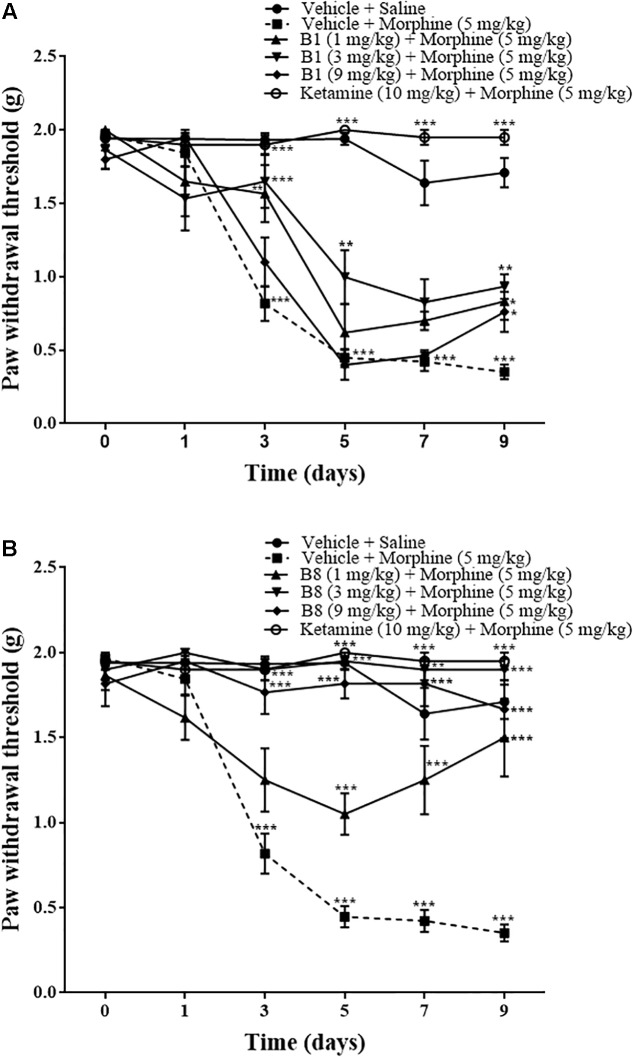
Effects of benzimidazole derivatives, B1/B8, on paw withdrawal threshold during morphine-induced paradoxical pain in mice. Mice of either gender (*n* = 6–10) received intraperitoneal vehicle; vehicle+morphine (5 mg/kg); ketamine (10 mg/kg)+morphine; **(A)** B1 (1 mg/kg, 3 mg/kg or 9 mg/kg)+morphine or **(B)** B8 (1 mg/kg, 3 mg/kg or 9 mg/kg)+morphine injected twice daily for 6 days. Paw withdrawal threshold was measured prior to drug administration (naïve baseline), every alternate day (1st, 3rd, 5th, and 7th day at 08:30 h) during the drug treatment period 30 min after morphine/saline injection and 96 h (9th day) after the last IP drug injection by using von Frey filaments. Control animals received an equal volume of vehicle. Analyzed by two-way ANOVA followed by Tukey’s *post hoc* test. Data are expressed as mean ± SEM. ^∗^*p* < 0.05, ^∗∗^*p* < 0.01, ^∗∗∗^*p* < 0.001.

Repeated morphine treatment led to a gradual decrease in the paw withdrawal threshold. The decrease was significant starting from day 3 of morphine administration at 0.87 ± 0.11 g (^∗∗∗^*p* < 0.001), 0.44 ± 0.062 g (^∗∗∗^*p* < 0.001) on day 5, 0.422 ± 0.064 g (^∗∗∗^*p* < 0.001) on day 7, and 0.351 ± 0.05 g (^∗∗∗^*p* < 0.001) at 96 h after morphine withdrawal relative to the vehicle saline group (two-way ANOVA, *n* = 9) (Figure 4A,B). Mice receiving B1 (3 mg/kg) with morphine did not exhibit a significant decrease in mean paw withdrawal latency, with 1.65 ± 0.184 g (^∗∗∗^*p* < 0.001) on day 3, 0.99 ± 0.18 g (^∗∗^*p* < 0.01) on day 5, and 0.93 ± 0.08 g (^∗∗^*p* < 0.01) at 96 h after morphine withdrawal relative to the vehicle-morphine group (two-way ANOVA, *n* = 6) (Figure 4A). B1 (1 mg/kg) exhibited an increase in mean paw withdrawal latency of 1.566 ± 0.194 g (^∗∗^*p* < 0.01) on day 3 and 0.833 ± 0.128 g (^∗^*p* < 0.05) at 96 h after withdrawal, relative to the vehicle-morphine group (two-way ANOVA, *n* = 6) (Figure 4A). B1 (9 mg/kg) also showed an increase in mean paw withdrawal latency 0.76 ± 0.136 g (^∗^*p* < 0.05) at 96 h after morphine withdrawal relative to vehicle-morphine group (two-way ANOVA, *n* = 6) (Figure 4A). Mice receiving B8 (1 mg/kg, 3 mg/kg and 9 mg/kg) with morphine did not exhibit a significant decrease in mean paw withdrawal latency, indicating that B8 treatment attenuates the development of mechanical allodynia due to repeated morphine treatment. The mean paw withdrawal latency of B8 (3 mg/kg) or B8 (9 mg/kg) was 1.9 ± 0.063 g (^∗∗∗^*p* < 0.001) or 1.7 ± 0.12 g (^∗∗∗^*p* < 0.001) on day 3, 1.95 ± 0.05 g (^∗∗∗^*p* < 0.001) or 1.81 ± 0.08 g (^∗∗∗^*p* < 0.001) on day 5, 1.9 ± 0.063 g (^∗∗∗^*p* < 0.001) or 1.81 ± 0.132 g (^∗∗∗^*p* < 0.001) on day 7, and 1.9 ± 0.063 g (^∗∗∗^*p* < 0.001) or 1.66 ± 0.17 g (^∗∗∗^*p* < 0.001) at 96 h after withdrawal, relative to the vehicle-morphine group (two-way ANOVA, *n* = 6) (Figure 4B). The mean paw withdrawal latency of B8 (1 mg/kg) was 1.05 ± 0.12 g (^∗∗∗^*p* < 0.001) on day 5, 1.2 ± 0.22 g (^∗∗^*p* < 0.01) on day 7, and 1.5 ± 0.22 g (^∗∗∗^*p* < 0.001) at 96 h after withdrawal, relative to the vehicle-morphine group (two-way ANOVA, *n* = 6) (Figure 4B). Administration of ketamine (positive control group) with morphine also did not show a significant decrease in mean paw withdrawal latency at 1.9 ± 0.063 g (^∗∗∗^*p* < 0.001) on day 3, 2 ± 0 g (^∗∗∗^*p* < 0.001) on day 5, 1.95 ± 0.05 g (^∗∗∗^*p* < 0.001) on day 7, and 1.95 ± 0.05 g (^∗∗∗^*p* < 0.001) at 96 h after withdrawal, relative to the vehicle-morphine group (two-way ANOVA, *n* = 6) (Figure 4A,B). These data showed that treatment with morphine on day 1 to day 6 results in an increase in tactile sensitivity compared with the control. Moreover, co-administration of benzimidazole derivatives reduced the allodynic effect of repeated morphine administration.

### Effect of Benzimidazole Derivatives on Repeated Morphine-Mediated Thermal Nociceptive Test

**FIGURE 5:**
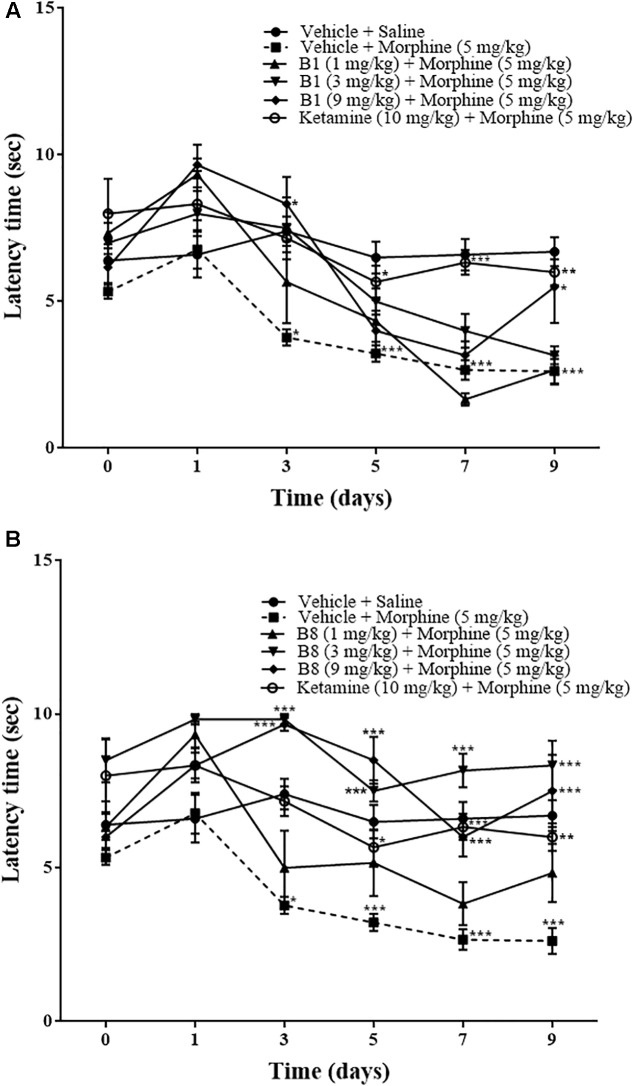
Effects of benzimidazole derivative, B1/B8, on tail latency with thermal nociception during morphine-induced paradoxical pain in mice. Mice of either gender (*n* = 6–10) received intraperitoneal vehicle; vehicle+morphine (5 mg/kg); ketamine (10 mg/kg)+morphine; **(A)** B1 (1 mg/kg, 3 mg/kg or 9 mg/kg)+morphine or **(B)** B8 (1 mg/kg, 3 mg/kg or 9 mg/kg)+morphine injected twice daily for 6 days. Thermal pain sensitivity was measured prior to drug administration (naïve baseline) every other day (1st, 3rd, 5th, and 7th day at 08:30 h) during the drug treatment period 30 min after morphine/saline injection and 96 h (9th day) after the last IP drug injection by using water bath (45°C ± 1°C). Control animals received an equal volume of vehicle. Analyzed by two-way ANOVA followed by Tukey’s *post hoc* test. Data are expressed as mean ± SEM. ^∗^*p* < 0.0, ^∗∗^*p* < 0.01, ^∗∗∗^*p* < 0.001.

Repeated morphine treatment led to the development of significant increase in thermal pain sensitivity. The increase in pain sensitivity was 3.77 ± 0.27 s (^∗^*p* < 0.05) on day 3 relative to the vehicle-saline group (two-way ANOVA *n* = 9). Mean latency time of the vehicle-morphine group was 3.22 ± 0.27 s (^∗∗∗^*p* < 0.001) on day 5, 2.66 ± 0.33 s (^∗∗∗^*p* < 0.001) on day 7, and 2.77 ± 0.406 s (^∗∗∗^*p* < 0.001) at 96 h after withdrawal, relative to the vehicle-saline group (two way-ANOVA. *n* = 9) (Figure 5A,B). Mice receiving B1 (9 mg/kg) showed an increase in latency time. The mean latency time of B1 (9 mg/kg) was 8.66 ± 1.45 s (^∗^*p* < 0.05) on day 3 and 5.5 ± 1.23 s (^∗^*p* < 0.05) at 96 h after withdrawal, relative to the vehicle-morphine group (two-way ANOVA, *n* = 6) (Figure 5A). Administration of B8 with morphine showed an increase in latency time. The mean latency times of the B8 (3 mg/kg) and B8 (9 mg/kg) were 10 ± 0.33 s (^∗∗∗^*p* < 0.001) or 9.5 ± 0.42 s (^∗∗∗^*p* < 0.001) on day 3, 7.5 ± 0.34 s (^∗∗∗^*p* < 0.001) or 8.66 ± 0.84 s (^∗∗∗^*p* < 0.001) on day 5, 8.16 ± 0.79 s (^∗∗∗^*p* < 0.001) or 6 ± 0.63 s (^∗∗∗^*p* < 0.001) on day 7, and 8.3 ± 0.80 s (^∗∗∗^*p* < 0.001) or 7.5 ± 1.1 s (^∗∗∗^*p* < 0.001) at 96 h after withdrawal, relative to vehicle-morphine group (two-way ANOVA, *n* = 6) (Figure 5B). Administration of the vehicle-ketamine positive control group also showed a significant increase in latency time at 5.66 ± 0.55 s (^∗^*p* < 0.05) on day 5, 6.33 ± 0.421 s (^∗∗∗^*p* < 0.001) on day 7, and 6 ± 0.44 s (^∗∗^*p* < 0.01) at 96 h after withdrawal, relative to vehicle-morphine group (two-way ANOVA, *n* = 6) (Figure 5A,B). The results showed that on the first day of testing, all groups exhibited comparable baseline tail flick latencies. Repeated IP administration of morphine across time resulted in a reduction of tail flick response, indicating an increased sensitivity to painful stimuli. However, co-administration of benzimidazole derivatives reduced tail flick latencies across these treatment periods.

### Effect of Benzimidazole Derivative on TNF-α in Morphine Treated-Mice

**FIGURE 6:**
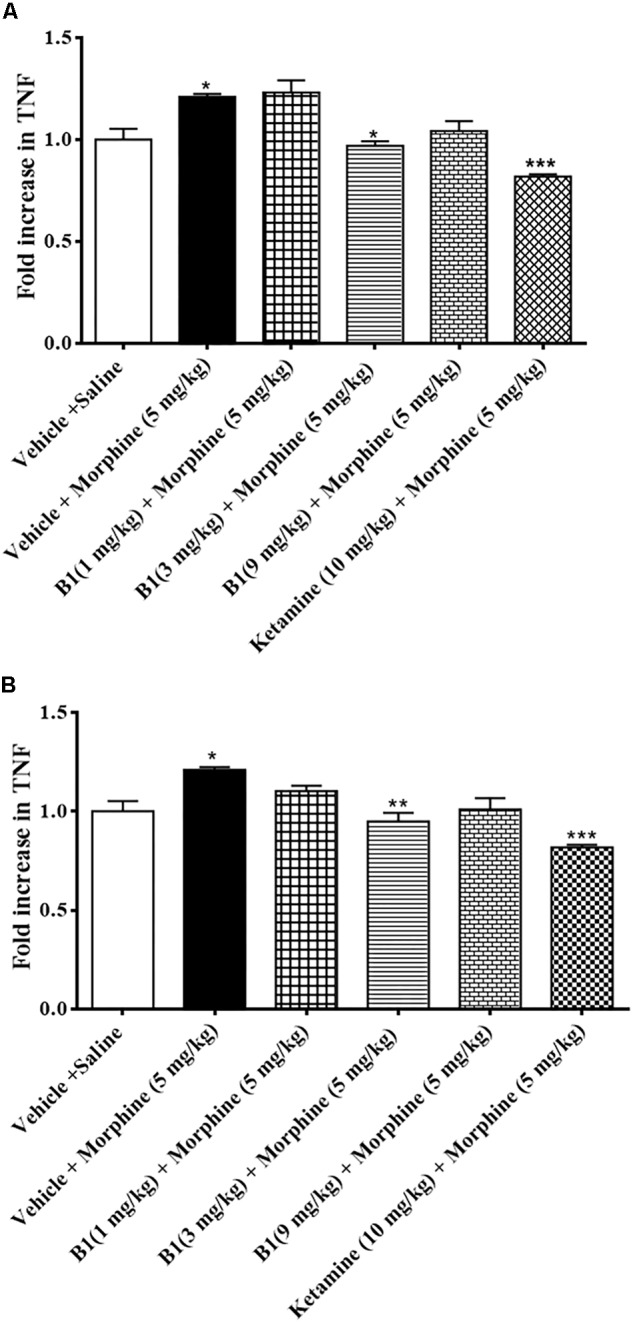
Effects of benzimidazole derivative, B1/B8, on spinal TNF-α expression during morphine withdrawal in mice. **(A,B)** At 96 h after the last dose of morphine, the animals were sacrificed after performing behavioral testing; spinal cords were isolated and stored at –80°C. The spinal cord was homogenized as described in the method section and the TNF-α levels were measured by ELISA assay. ^∗^*p* < 0.05, ^∗∗^*p* < 0.01, ^∗∗∗^*p* < 0.001. Analyzed by one-way ANOVA followed by Tukey’s *post hoc* test.

Spinal cord tissue isolated from the morphine-administrated animal group showed an increase in TNF-α expression of 1.2 ± 50.01 pg/mg protein (^∗^*p* < 0.05) compared to saline group (*n* = 3 per group) (Figure 6A,B) (one-way ANOVA). B1 (3 mg/kg) resulted in a decrease in TNF-α expression of 0.96 ± 0.02 pg/mg protein (^∗^*p* < 0.05) corresponding to morphine-treated mice (*n* = 3 per group) (Figure 6A) (one-way ANOVA). Meanwhile, administration of B8 (3 mg/kg) also decreased TNF-α expression, at 0.94 ± 0.04 pg/mg protein (^∗∗^*p* < 0.01) corresponding to morphine-treated mice (*n* = 3 per group) (Figure 6B) (one-way ANOVA). Administration of ketamine also showed a decrease in TNF-α expression of 81.81 ± 1.22 pg/mg protein (^∗∗∗^*p* < 0.001) corresponding to morphine-treated mice (*n* = 3 per group (Figure 6A,B) (one-way ANOVA).

### Antioxidant Effect

The result of concentration-dependent free radical scavenging activity of B1, B8 and ascorbic acid are shown in (Table 1). The IC_50_ was calculated for each compound. B1 showed a radical scavenging affect, with an IC_50_ value of 293 μg/ml. The B8 possesses less antioxidant potential, with an IC_50_ value of more than 1000 μg/ml. Ascorbic acid showed its antioxidant potential with an IC_50_ value of 241 μg/ml.

### Acute Toxicity in Animal Model

All mice survived and no change in animals’ behavior was observed after 24 h of B1 and B8 administration (data not shown).

## Discussion

The present study demonstrates that administration of benzimidazole derivatives, B1 and B8, attenuated morphine-induced hyperalgesia and allodynia (Figure 3–5) and decreased the expression of TNF-α (Figure 6). The study also showed that paradoxical pain decreases the analgesic action of morphine (Figure 3–5). Previous studies (Nichols et al., 1997) and the present results indicate that an increase in pain threshold latency against noxious mechanical and thermal stimuli in morphine-treated mice indicates the analgesic effect of benzimidazole derivatives, involving reduced TNF-α expression.

The mechanism underlying the sustained morphine-mediated paradoxical pain including hyperalgesia and allodynia is not entirely clear. Opioid-induced hyperalgesia and allodynia in mice suggest that cross-interaction between hypersensitivity mechanisms exists during the process of neuropathic pain and continued opioid treatment (Raghavendra et al., 2002). It has been postulated that central neuro-immune activation and neuro-inflammation contributes to the persistent pain mechanism (DeLeo and Yezierski, 2001; Milligan and Watkins, 2009).

The CNS glial cells play an important part in the amplification of neuronal pain. Glial cells are activated after nerve injury through a specific signal detected by microglial cells. Activated microglial cells release multiple proinflammatory mediators in the spinal cord. It has been reported that in neuropathic pain, either due to nerve damage or peripheral inflammation, pro-inflammatory mediators such as TNF-α increase in the spinal cord (DeLeo and Yezierski, 2001). Released spinal pro-inflammatory mediators (Ji and Strichartz, 2004) increase the excitability of primary neurons in response to external stimuli (Gardell et al., 2002). Chronic morphine treatment elevates TNF-α concentrations (Raghavendra et al., 2002) in neuropathic pain. Previous studies showed that central or peripheral administration of TNF-α induces hyperalgesia and allodynia in rodents (Peterson et al., 1998) and postulated that cytokines might interact with opioid receptors and modulate their actions (Raghavendra et al., 2004). Increased pro-inflammatory mediators in sustained morphine-treated mice increase the behavioral hypersensitivity to noxious and non-noxious stimuli observed during the morphine administration period (Figure 3–5). Furthermore, sustained opioid treatment activates glial cells indirectly by stimulating excitatory neurotransmitter release from the central termini of the primary sensory neurons and/or from second-order spinal neurons or by inhibiting descending facilitatory mechanisms (Ossipov et al., 2005). In line with these studies, the present study revealed that pro-inflammatory mediators were elevated in the spinal cord of sustained morphine-treated mice, as evidenced by increased TNF-α concentrations in the spinal cord.

It has been reported that N-methyl-D-aspartate (NMDA) signaling might be involved in the pain amplification mechanism of morphine-treated mice. Glutamate is released from primary afferent neurons in response to acute and more persistent chronic pain. Glutamate, the excitatory amino acid, produces its effect through the NMDA receptor (Basbaum et al., 2009). Activation of the NMDA receptor leads to elevated responsiveness and increased activity of dorsal horn neurons that cause central sensitization such as tactile allodynia and hyperalgesia (Basbaum et al., 2009). Thus, the NMDA pathway enhances spinal mechanisms in tissue damage and has an important role both in the induction and maintenance of pain. The inhibition of the NMDA pathway with ketamine, an NMDA antagonist, was explored as an analgesic activity in great depth (D’Mello and Dickenson, 2008). In the current study, that administration of ketamine (10 mg/kg) substantially decreased morphine-induced hyperalgesia and allodynia in sustained morphine-treated mice suggests that the opioids may elicit NMDA-dependent pain hypersensitivity (Figure 3–5). This is due to the fact that there is a widespread distribution of NMDA receptors, so the administration of antagonist will not only target pathology but will also affect the normal essential NMDA signaling. In this way, patients taking NMDA antagonist are associated with adverse effects (D’Mello and Dickenson, 2008).

To increase the efficacy of opioids, inhibition of glial cell activity might be effective (Song and Zhao, 2001). Unfortunately, currently available “classical” glial pro-inflammatory inhibitors are either toxic (fluorocitrate) or are not specific for glia (minocycline and propentofylline) (Raghavendra et al., 2002). So, potentially more demanding and selective methods are required to modulate glial cell activity.

Peroxisome proliferator-activated receptor gamma (PPARγ) belongs to a family of nuclear receptors. PPARγ has been expressed in the cells of monocytes or macrophage lineages inclusive of brain microglial cells. PPARγ inhibits microglial cell action by impeding the expression of a number of PPARγ-regulated genes that become elevated during the cellular process (Breidert et al., 2002). Mediators that were prevented by PPARγ ligands were proinflammatory cytokines such as TNF-α (Breidert et al., 2002). Recent research indicates that PPARγ-mediated inhibition of glial cell activation plays an important part in their efficacy in neuropathic pain. Therefore, we hypothesized that glial cell PPARγ agonists might be able to inhibit morphine-mediated spinal glial cell activation and thus arrest paradoxical pain.

Benzimidazole derivatives, B1 or B8, are promising and have interesting therapeutic potential for pain management. Benzimidazole moieties accomplish the possible structural specifications that are necessary for anti-inflammatory activity along with analgesic potential (Akhtar et al., 2017). It was also suggested that the analgesic effects of the benzimidazole derivative might possibly be due to PPARγ-mediated inhibition of glial cells. The effect of benzimidazole derivatives were studied by *in vivo* and *in vitro* methods. B1 (1 or 9 mg/kg) increases the reaction time to the hot plate test and (9 mg/kg) increases latency time to thermal nociception. von Frey filament test results showed that B1 increases the paw withdrawal threshold at all three doses (1, 3, or 9 mg/kg) while B8 (1, 3, or 9 mg/kg) increases the reaction time to hot plate, latency time to thermal nociception and paw withdrawal threshold. All these results show that B8 is more effective at all three doses compared to B1. Benzimidazole derivatives B1 or B8 (3 mg/kg), reduce TNF-α expression in the lumbar spinal cord of the morphine-treated mice in ELISA, indicating a reduction in proinflammatory contents.

Taken together, these data suggest that administration of benzimidazole derivatives B1 and B8 may attenuate the neuro-inflammatory consequence of long-term opioid agonist therapy as it attenuates the upregulation of TNF-α in the lumber spinal cord of morphine-withdrawn mice (Figure 6), indicating that benzimidazole derivatives’ administration may be an effective method to reduce the pro-inflammatory consequence of sustained opioid analgesic treatment.

The *in vitro* antioxidant activities of the benzimidazole derivatives were also investigated by DPPH free radical scavenging assay. Antioxidants from DPPH free radical scavenging are due to their hydrogen donating ability. The antioxidant potential of the selected compounds was ascorbic acid> compound B1 > compound B8 on the basis of their calculated IC_50_ value (Table 1).

**Table 1:** Antioxidant activity evaluation by DPPH method (mean ± SEM).

Concentration (μg/ml)	Percentage of inhibition		
	B1+DPPH	B8+DPPH	Ascorbic acid
1	2.039 ± 0.48	1.31 ± 0.14	2.16 ± 0.18
3	6.07 ± 2.79	2.10 ± 0.43	4.31 ± 0.45
10	7.95 ± 3.38	4.16 ± 0.01	4.23 ± 0.33
100	24.96 ± 0.08	7.36 ± 0.44	15.95 ± 0.72
300	50.78 ± 0.59	7.49 ± 2.32	64.18 ± 0.13
700	62.96 ± 0.51	10.24 ± 2.56	92.22 ± 0.01
1000	65.59 ± 1.23	18.36 ± 0.006	93.29 ± 0.09
Blank (negative control)	3.99	3.99	3.34
IC50	293 μg/ml	More than 1000 μg/ml	241 μg/ml

The acute toxicity showed the safety of benzimidazole derivatives, as no mortality or other significant gross behavioral changes were observed at 1000 mg/kg, which showed the safety of benzimidazole derivatives up to 1000 mg/kg.

It was reported that repeated opioid administration leads to paradoxical pain sensitivity, resulting in a need to intensify opioid doses during the treatment duration of chronic pain (Vanderah et al., 2001). However, elevated doses exaggerate the side effects of opioids such as constipation, respiratory depression and addiction liability. Our present results anticipate a unique pharmacological prospective that the benzimidazole derivatives attentuate morphine-induced paradoxical pain and neuro-inflammatory outcomes. Benzimidazole derivatives increase both the efficacy and duration of action of the opioid analgesics, which might offer them as novel therapeutic agents for opioid-induced paradoxical pain.

## Conclusion

In conclusion, the present study clearly demonstrates that benzimidazole derivatives: *N*-[(1*H*-benzimidazol-2-yl)methyl]-4-methoxyaniline, *N*-{4-[1*H*-benzimidazol-2-yl)methoxy]phenyl} acetamide exhibit analgesic effects due to PPARγ agonist activity, which indicates their therapeutic prospective in morphine-induced paradoxical pain. However, further studies are warranted to conduct pharmacokinetics and extensive toxicity studies of these compounds. It is also suggested that further research on underlying principles of neuro-immune pathways of opioid-induced paradoxical pain is required that would improve the clinical management of opioid therapy and also in the management of chronic pain syndromes.

## Ethics Statement

The research study was approved by Research and Ethical Committee at Riphah Institute of Pharmaceutical Sciences, Riphah International University, Islamabad and reference number is REC/RIPS/2017/014.

## Author Contributions

ZI carried out the experimental work under the supervision of MA. A-uK worked as co-supervisor. HN synthesized the compounds.

## Conflict of Interest Statement

The authors declare that the research was conducted in the absence of any commercial or financial relationships that could be construed as a potential conflict of interest.
